# Supplementary material for: To be or not to be a nonhost species: A case study of the Leptosphaeria maculans and Brassica carinata interaction
Source: Environ Microbiol Rep. 2024 Nov 28;16(6):e70034. doi: 10.1111/1758-2229.70034 (PMC11603210; doi:10.1111/1758-2229.70034)

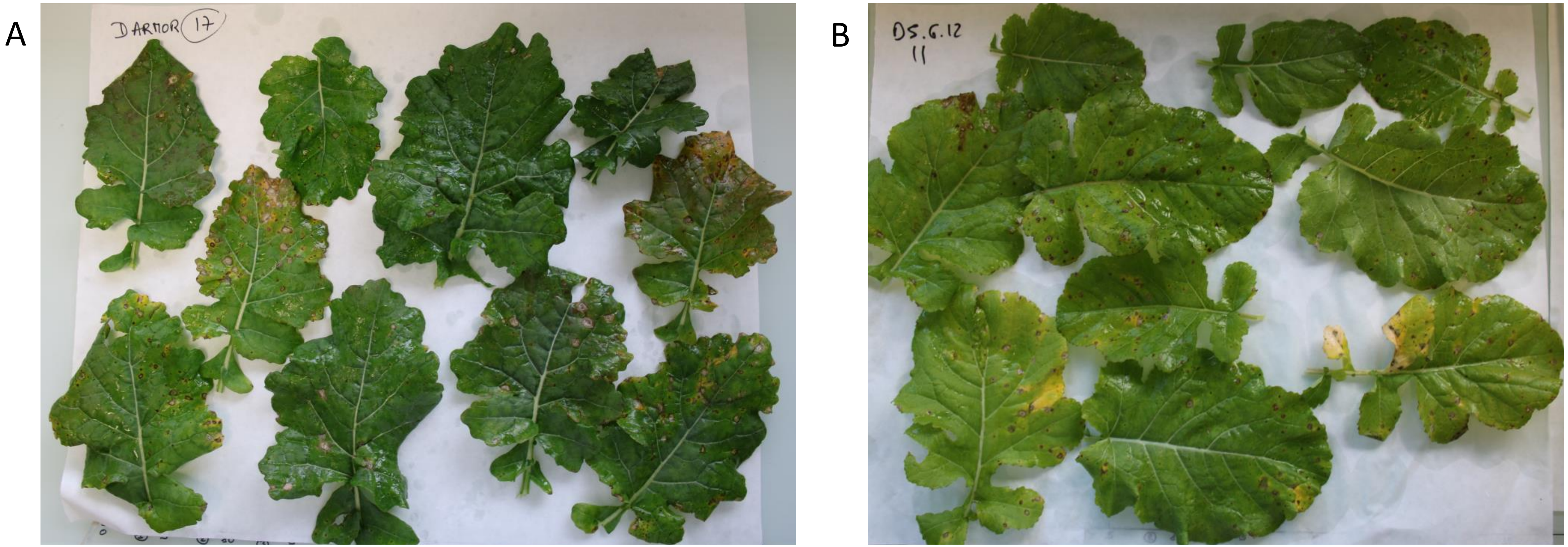

Figure S1. Symptoms recovered on leaves of *Brassica napus* (cv. Darmor) and *Brassica carinata* (line D5.6.12) in a field experiment. Leaves of (A) *B. napus* cv. Darmor and (B) *B. carinata* line D5.6.12. Examples of symptoms observed on leaf disks collected 2.5 months after sowing on (C), (D) *B. napus* and (E), (F) *B. carinata*. Symptoms identified from disks (C), and (E) were suspected to be caused by *L. biglobosa*; while (D) and (F) were suspected to be caused by *L. maculans*.

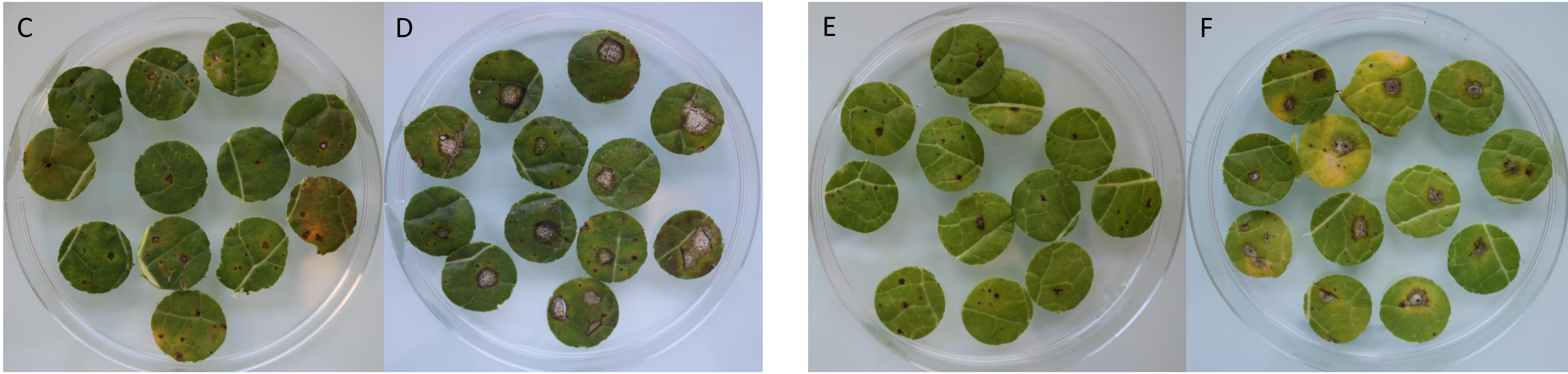

Supplement: Supplementary file 1 — FIGURE S1. Symptoms recovered on leaves of Brassica napus (cv. Darmor) and Brassica carinata (line D5.6.12) in a field experiment. Leaves of (A) B. napus cv. Darmor and (B) B. carinata line D5.6.12. Examples of symptoms observed on leaf disks collected 2.5 months after sowing on (C), (D) B. napus and (E), (F) B. carinata. Symptoms identified from disks (C), and (E) were suspected to be caused by L. biglobosa; while (D) and (F) were suspected to be caused by L. maculans. [file EMI4-16-e70034-s009.pdf]
