# Supplementary material for: To be or not to be a nonhost species: A case study of the Leptosphaeria maculans and Brassica carinata interaction
Source: Environ Microbiol Rep. 2024 Nov 28;16(6):e70034. doi: 10.1111/1758-2229.70034 (PMC11603210; doi:10.1111/1758-2229.70034)
Supplement: Supplementary file 4 — FIGURE S4. Symptoms observed on Brassica carinata line D5.6.12 following brush inoculation with isolate V77.1.11 of Leptosphaeria maculans. The upper line displays details of the experiment; with, from left to right; JS_2018_3, the reference of the inoculation; the dates of start and end of the experiment (i.e., dates of inoculation and observation); AF425, the reference of the B. carinata line used for the inoculation (i.e., line D5.6.12); Line 3, the line of the plants used for inoculation; V77.1.11, the name of the L. maculans isolate brush‐inoculated on the cotyledons (Section 2). [file EMI4-16-e70034-s011.pdf]

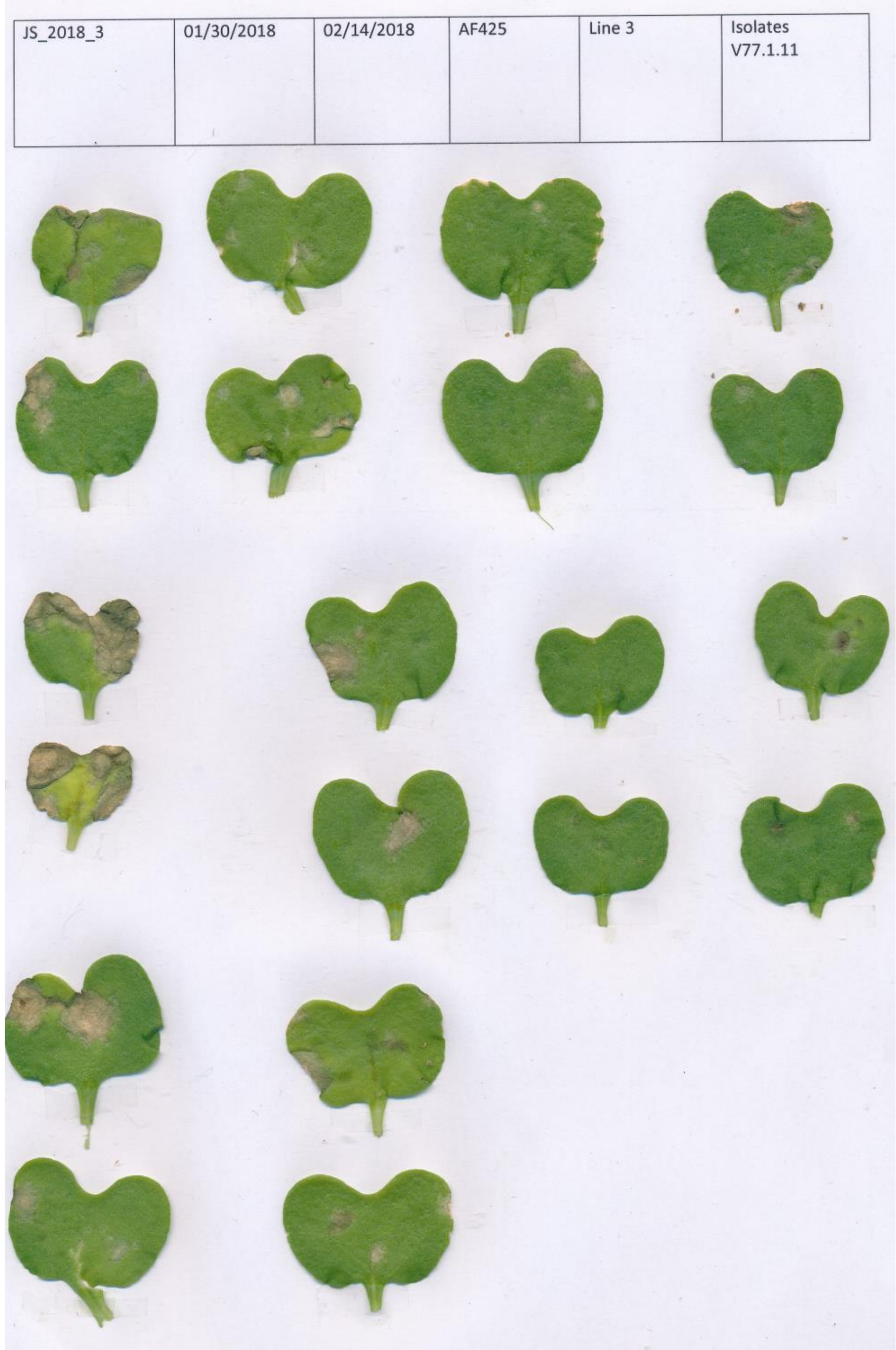

Figure S4. Symptoms observed on *Brassica carinata* line D5.6.12 following brush inoculation with isolate V77.1.11 of *Leptosphaeria maculans*. The upper line displays details of the experiment; with, from left to right; JS_2018_3, the reference of the inoculation; the dates of start and end of the experiment (i.e. dates of inoculation and observation); AF425, the reference of the *B. carinata* line used for the inoculation (i.e. line D5.6.12); Line 3, the line of the plants used for inoculation; V77.1.11, the name of the *L. maculans* isolate brush-inoculated on the cotyledons (Experimental Procedures).
