# Supplementary material for: To be or not to be a nonhost species: A case study of the Leptosphaeria maculans and Brassica carinata interaction
Source: Environ Microbiol Rep. 2024 Nov 28;16(6):e70034. doi: 10.1111/1758-2229.70034 (PMC11603210; doi:10.1111/1758-2229.70034)
Supplement: Supplementary file 5 — FIGURE S5. Symptoms observed on Brassica napus cv. Yudal following brush inoculation with the isolate JN2 of Leptosphaeria maculans. The upper line displays details of the experiment; with, from left to right; JS_2018_3, the reference of the inoculation; the dates of start and end of the experiment (i.e., dates of inoculation and observation); Yudal, the reference of the B. napus cultivar used for the inoculation; Line 4, the line of the plants used for inoculation; JN2, the name of the L. maculans isolate brush‐inoculated on the cotyledons (Section 2). [file EMI4-16-e70034-s003.pdf]

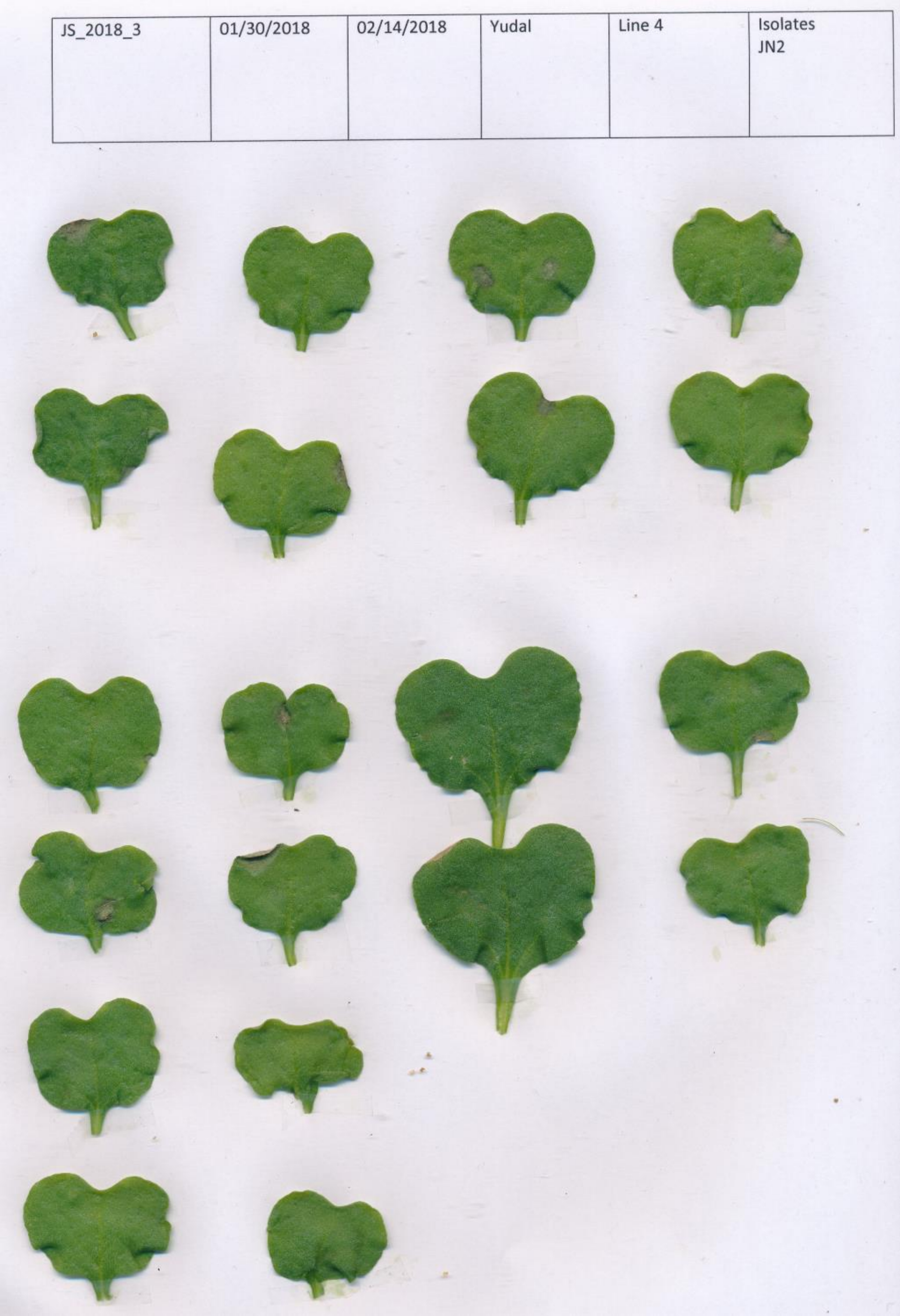

Figure S5. Symptoms observed on *Brassica napus* cv. Yudal following brush inoculation with the isolate JN2 of *Leptosphaeria maculans*. The upper line displays details of the experiment; with, from left to right; JS_2018_3, the reference of the inoculation; the dates of start and end of the experiment (i.e. dates of inoculation and observation); Yudal, the reference of the *B. napus* cultivar used for the inoculation; Line 4, the line of the plants used for inoculation; JN2, the name of the *L. maculans* isolate brush-inoculated on the cotyledons (Experimental Procedures).
