# Supplementary material for: To be or not to be a nonhost species: A case study of the Leptosphaeria maculans and Brassica carinata interaction
Source: Environ Microbiol Rep. 2024 Nov 28;16(6):e70034. doi: 10.1111/1758-2229.70034 (PMC11603210; doi:10.1111/1758-2229.70034)
Supplement: Supplementary file 8 — FIGURE S8. Internal necrosis caused by three Leptosphaeria maculans isolates on stems of Brassica napus and Brassica carinata. Transversal sections of stems of B. napus, upper panel, and B. carinata, lower panel; (A) and (E) uninfected stems; (B) and (F) JN3‐infected stems; (C) and (G) HB10.19‐infected stems; (D) and (H) V77.1.11‐infected stems. Uninfected stems were used as a negative control. [file EMI4-16-e70034-s007.pdf]

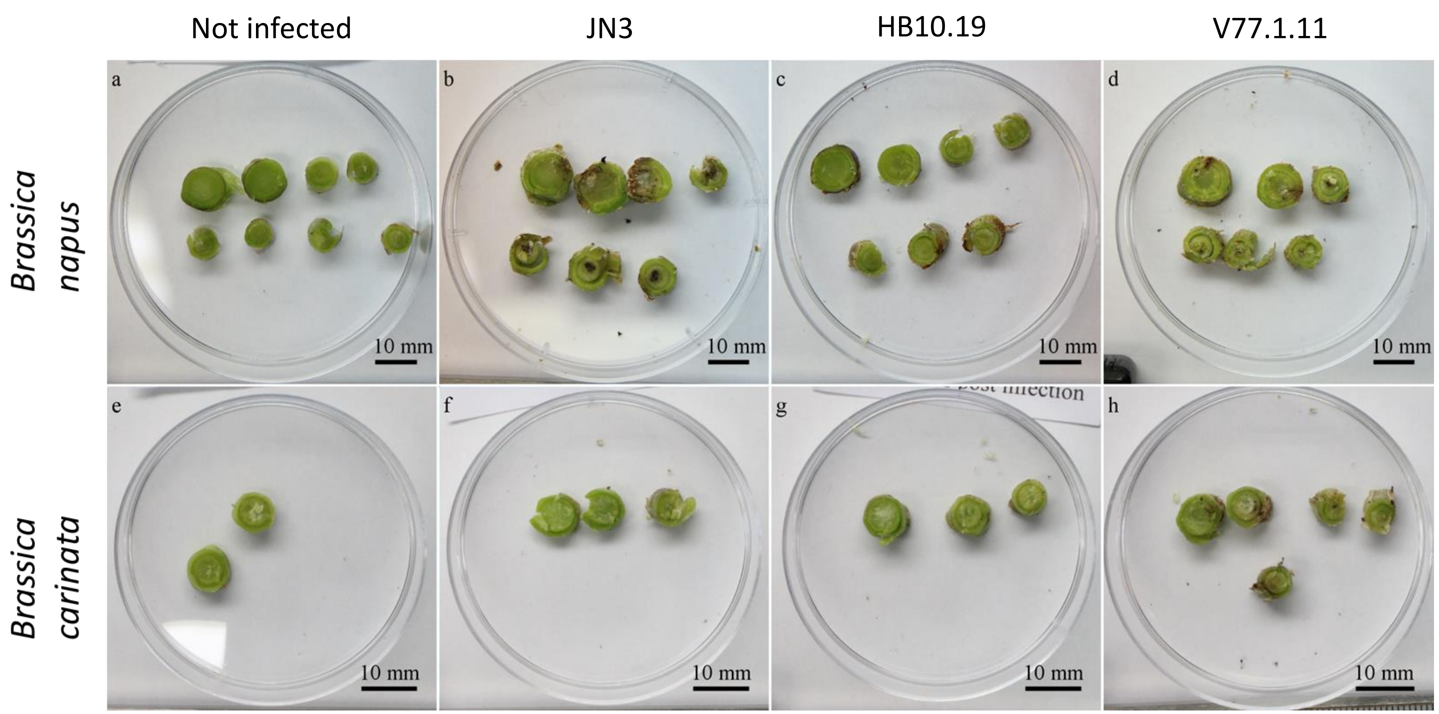

Figure S8. Internal necrosis caused by three *Leptosphaeria maculans* isolates on stems of *Brassica napus* and *Brassica carinata*. Transversal sections of stems of *B. napus*, upper panel, and *B. carinata*, lower panel; a) and e) uninfected stems; b) and f) JN3-infected stems; c) and g) HB10.19-infected stems; d) and h) V77.1.11-infected stems. Uninfected stems were used as a negative control.
